# Quantifying and Utilizing Electroosmotic Flow for Mechanical Measurements with the Scanning Ion Conductance Microscope

**DOI:** 10.1021/acs.analchem.5c03186

**Published:** 2025-10-03

**Authors:** Johannes Rheinlaender, Tilman E. Schäffer

**Affiliations:** Institute of Applied Physics, 9188 University of Tübingen, Auf der Morgenstelle 10, 72076 Tübingen, Germany

## Abstract

The scanning ion conductance microscope (SICM) is an emerging imaging technique for the investigation of delicate samples on the nanometer scale in liquid environments using ion current through a glass nanopipette. In recent years, the SICM has been increasingly applied to mechanical measurements, typically using a microfluidic flow in the nanopipette induced by hydrostatic pressure. Here, we introduce the use of electroosmotic flow (EOF) in mechanical SICM measurements. We show that the EOF in small SICM nanopipettes is comparable to the flow induced by commonly applied hydrostatic pressures. We quantify the electroosmotic mobility, which is a central parameter of EOF but strongly depends on experimental conditions, by measuring the streaming current independent of nanopipette geometry. Using decane microdroplets, we show that both EOF and hydrostatic pressure can be used to mechanically probe elastic samples on the nanometer scale. We then develop a numerical model to quantify the stiffness and the Young’s modulus of elastic samples using EOF. Finally, we use EOF to map the Young’s modulus of living cells, which gives similar results to the hydrostatic pressure method. We thereby demonstrate that EOF can be used to quantitatively probe sample stiffness with the SICM.

## Graphical abstract

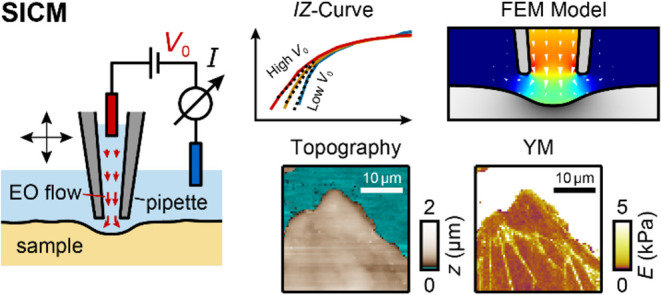

## Introduction

The scanning ion conductance microscope (SICM) was developed in the late 90′s.^1^ It benefits from a contact-free imaging principle based on measuring the ion current through a fine nanopipette, which makes it especially useful for imaging of soft, delicate samples such as living cells,^2–4^ suspended membranes,^5^ or liquid–liquid interfaces.^6,7^ Applications of SICM include combinations with electrophysiology,^8,9^ local pH^10^ or chemical^11^ sensing, or local electrochemical,^12,13^ molecular,^14,15^ or liquid^16,17^ deposition. Moreover, a SICM nanopipette was used to mechanically stimulate^18,19^ or probe samples,^20–22^ which was achieved by creating a microfluidic flow via a hydrostatic pressure applied to the upper, macroscopic end of the nanopipette.^20^

An aspect rarely considered in SICM experiments is electroosmosis (EO) in the nanopipette.^23^ Electroosmotic flow (EOF) is the movement of liquid due to the force exerted by an external electric field on the counterions in the vicinity of a charged surface and is relevant in microfluidics and nanofluidics.^24,25^ However, despite various implications of EOF in biological and synthetic nanopores and numerous technological applications,^26^ many aspects of EO in nanopores are still under investigation, for example, the complex interaction with current rectification,^27^ flow rectification,^28^ or nonlinear flow patterns.^29^ In the context of the SICM, it has been speculated that EOF may influence molecular deposition^30^ and delivery,^31^ nanoparticle translocation,^32^ and ion current fluctuations.^33^

However, the mechanical effects of EOF in SICM experiments have not been investigated yet. This study therefore focuses on the quantification of EOF in SICM nanopipettes and the implications for measurements of sample mechanics. We demonstrate analytically and experimentally that EOF and hence the resulting force on the surface becomes relevant and comparable to the flow and forces induced by hydrostatic pressure for SICM glass nanopipettes with opening radii below 100 nm and/or applied voltages above 1 V and hence can be used for mechanical SICM measurements. However, the EO mobility strongly depends on the experimental conditions,^34^ which can vary widely between SICM applications. We therefore measure the streaming current, which is caused by the flow due to an externally applied pressure,^35^ to quantify the EO mobility in a running SICM experiment. To account for the complex nanopipette-sample geometry, we introduce a numerical model to quantitatively determine the stiffness and Young’s modulus of soft samples with the SICM using EOF. In conclusion, we show that EOF can have a considerable effect in SICM glass nanopipettes and provides a complementary approach to mechanical measurements with the SICM.

## Experimental Section

### SICM Setup

A home-built SICM head in combination with a commercial AFM setup (Figure 1a, for details see^36^) was used for all measurements. The SICM setup was combined with a commercial Nikon Ti–U inverse widefield fluorescence microscope. Measurements were recorded at room temperature or at 37 °C (U2OS cells, using a custom-built sample heater, for details see^36^). A voltage between *V*_0_ = 100 and 5000 mV was applied to the nanopipette using Ag/AgCl half-cells as electrodes, resulting in an ion current *I* of typically a few nanoamperes, depending on *V*_0_ and pipette size, practically limited by the measurement range of the ion current amplifier (here 20 nA). For measuring streaming current and sample stiffness based on hydrostatic pressure for comparison, an air pressure *p*_0_ of up to ca. 100 kPa was applied to the upper end of the nanopipette using a pressure regulator or a syringe. *IZ*-curves were recorded with typically 3 μm distance and 20 Hz rate and a relative current drop of 2% on an *xy* raster pattern as described before.^22^ Data were recorded and analyzed in Igor Pro (WaveMetrics Inc., Lake Oswego, OR).

**Figure 1:**
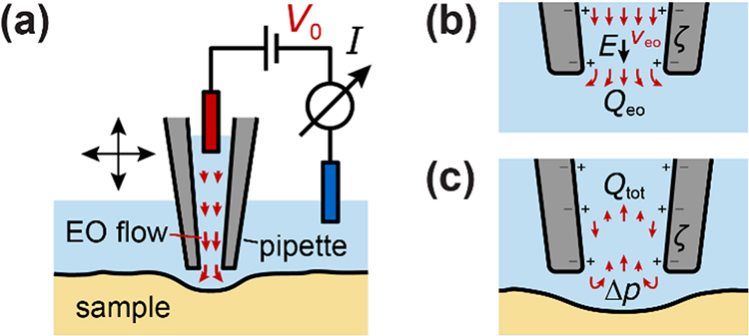
The EOF in the SICM. (a) Setup schematic with a nanopipette with applied voltage *V*_0_, resulting in an ion current *I* and inducing an EOF through the nanopipette potentially deforming the sample surface, and additionally applied hydrostatic pressure *p*_0_. (b) Schematic cross section of the nanopipette with (here negative) surface charge and ζ potential and (hence positive) counterions and electric field *E*, resulting in an EO velocity *v*_eo_ and a flow rate *Q*_eo_ far away from the sample. (c) Close to the sample, the EOF is partly blocked, resulting in a backflow and hence reduced total flow *Q*_tot_ and a pressure increase Δ*p* on the sample surface below the nanopipette.

### Nanopipettes

Nanopipettes were made from capillaries of borosilicate glass (1B100F-4, World Precision Instruments Inc., Sarasota, FL) using a commercial micropipette puller (P-2000, Sutter Instruments, Novato, CA). The pipets’ inner opening radius *r*_i_ was between 50–500 nm, depending on the puller program. The ratio of the outer to the inner nanopipette opening radius *r*_o_/*r*_i_ was typically 1.5 and the pipet inner half cone angle was typically α = 4° as determined by fitting *IZ*-curves.^37^ The nanopipettes were filled with either 100 mM NaCl (for decane droplets) with an electrical conductivity σ = 1.0 S/m at room temperature or with phosphate-buffered saline (PBS, D8537, Sigma-Aldrich, Taufkirchen, Germany) (for platelets and U2OS cells) with σ = 1.5 S/m at room temperature (platelets) and 2.0 S/m at 37 °C (U2OS cells).^38^

### Materials

To visualize the EO and pressure flow (Figure 2a), glass nanopipettes were filled with PBS containing 1 mM rhodamine B (R6626, Sigma-Aldrich). To change the surface charge of the glass (Figure 2b,c), nanopipettes were either filled with and immersed in PBS containing 1% (w/w) bovine serum albumin (BSA, A7906, Sigma-Aldrich) ca. 1 min before the measurement or coated with poly l-lysine (PPL) by filling the nanopipette tips with a few microliters of a 0.01% aqueous solution of PLL (P4707, Sigma-Aldrich) and drying for 10 min at 120 °C before filling with PBS. Decane droplets were fabricated by depositing 5–10 μL of *n*-decane (30550, Fluka Analytical, Buchs SG, Switzerland) on plastic bottom dishes (627161, Greiner Bio-One, Frickenhausen, Germany) and then immersing them with 100 mM NaCl as imaging solution. To reduce the interface tension and hence the droplet stiffness, Tween 20 (9127.1, Roth GmbH, Karlsruhe, Germany) was added to the imaging solution at a concentration of 0.03 mM, slightly above the critical micelle concentration.^7^ Living human platelets were prepared as described previously,^39^ allowed to adhere and spread on a culture dish (Cellstar, 627160, Greiner Bio-One) surface for approximately 10 min and imaged in PBS at room temperature. Living U2OS cells with RFP-tagged actin (CLL1038, Sigma-Aldrich) were cultured according to manufacturer specifications and seeded in culture dishes (Cellstar, 627160, Greiner Bio-One) for 24 h and imaged in L15 medium (21083027, Gibco, Thermo Fisher) at 37 °C.

**Figure 2:**
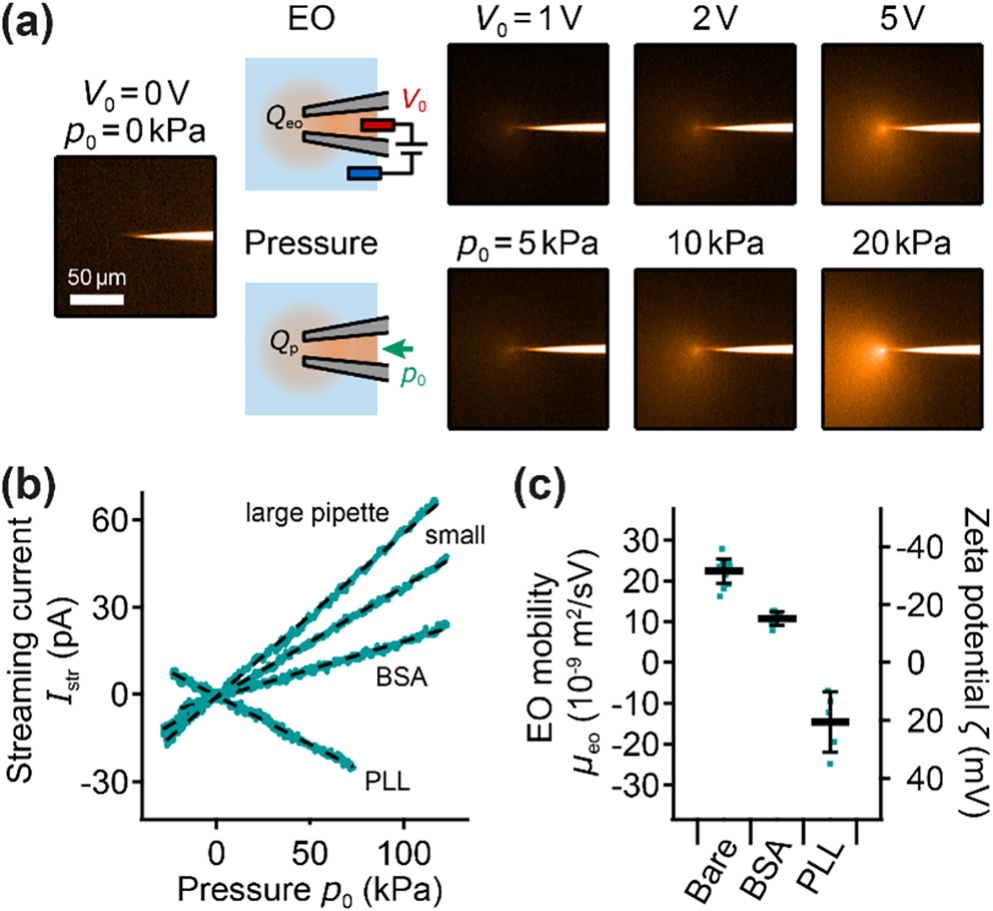
Quantification of EOF in a nanopipette and comparison with pressure flow. (a) Sideview fluorescent images and schematics of a nanopipette filled with a solution of rhodamine B and with different applied voltages *V*_0_ and pressures *p*_0_. (b) Streaming current *I*_str_ for a small and a large untreated (bare glass) nanopipette in PBS and for large nanopipettes treated with BSA and PLL with line fits (dashed traces). (c) EO mobility μ_eo_ and ζ potential measured from the streaming current for untreated nanopipettes and for nanopipettes treated with BSA or PLL. Bars and error bars show mean and standard deviation, respectively, of 5 to 14 individual nanopipettes per condition (markers).

### FEM Calculations

Finite element model (FEM) calculations were performed using a commercial software (COMSOL Multiphysics 4.1, COMSOL AB, Stockholm, Sweden) (see Figure S3a for details). Briefly, the nanopipette was modeled as conical with half opening angle α, ratio of outer to inner opening radius *r*_o_/*r*_i_ = 1.5, and axial length 1000*r*_i_ and placed within a distance *z* (in units of *r*_i_) above a linear-elastic sample with Young’s modulus *E*. A constant electric potential *V*_0_ and optionally a hydrostatic pressure *p*_0_ was applied to the upper end of the nanopipette relative to the bath solution. The electrolyte was modeled as an incompressible liquid with negligible density, viscosity η, electrical conductivity σ, and as electrically neutral, since surface charges are strongly screened at the electrolyte concentrations used here. The boundary condition at the surfaces of the nanopipette was set to constant electroosmotic mobility μ_eo_. To avoid unphysically large electric fields and hence flow velocities, the edges of the nanopipette opening were rounded to a radius of 0.1*r*_1_.

## Results and Discussion

### Basics of EOF in the SICM Nanopipette

In the SICM nanopipette, applying a positive voltage *V*_0_ to the nanopipette electrode relative to the bath electrode causes an outward EOF through the nanopipette (Figure 1a, red) due to the (usually) negatively charged glass surface of the (uncoated) nanopipette.^31^ When the nanopipette is far away from the sample surface, the EOF can freely exit the pipette opening, resulting in the typical uniform profile of ideal EOF inside the pipette with the velocity *v*_eo_ = *μ*_eo_·*E*, given by the electroosmotic mobility *μ*_eo_ and local electric field *E* (Figure 1b).^34^ *μ*_eo_ is proportional (but opposite in sign) to the ζ potential of the surface of the nanopipette with

1 $$\mu_{\mathrm{eo}}=-\frac{\varepsilon_{r}\varepsilon_{0}\zeta }{\eta }$$

where *η* is the viscosity of the electrolyte.^34^ Typically, *μ*_eo_ ≈ + 2·10^–8^ m^2^/(V s) for glass with *ζ* ≈ −20 mV and *η* ≈ 1.0 mPa·s for water-based electrolytes at physiological conditions.^31^ For free EOF (i.e., when the pipette is far from the surface), the volumetric flow rate *Q*_eo_ in a conical nanopipette with an inner opening radius *r*_i_, an inner half cone angle α, and an applied voltage *V*_0_ can be estimated as^31^

$$Q_{\mathrm{eo}}=\mu_{\mathrm{eo}}\cdot \pi r_{i}\tan\alpha \cdot V_{0}$$ 2

However, when the nanopipette is close to the sample (Figure 1c), the EOF is partially blocked by the sample, causing a pressure increase Δ*p* below the nanopipette and hence a pressure-induced backflow^20^

$$Q_{\mathrm{back}}=\frac{3\pi \tan\alpha }{8\eta }r_{i}^{3}\Delta p$$ 3

Assuming that the applied voltage *V*_0_ completely drops along the nanopipette and that the EOF is fully blocked by the sample and thus entirely compensated by the pressure-induced backflow, the total flow vanishes, *Q*_tot_ = *Q*_eo_ – *Q*_back_ = 0, giving the maximum theoretical EOF-induced pressure in a conical nanopipette

4 $$p_{\mathrm{eo}}≔\frac{8}{3}\frac{\mu_{\mathrm{eo}}\eta }{r_{i}^{2}}V_{0}$$

As expected, *p*_eo_ is proportional to the applied voltage *V*_0_, but, interestingly, inversely proportional to the square of the inner opening radius *r*_i_, and hence strongly increases for smaller nanopipettes. For example, for a typical SICM glass nanopipette with *r*_i_ = 100 nm and an applied voltage of *V*_0_ = 1 V, *p*_eo_ is on the order of 1 kPa, comparable to the typical hydrostatic pressures used in SICM.^40^

### Quantification of EOF in Nanopipettes

Since the EO mobility and effective ζ potential strongly depend on experimental conditions,^34^ we investigated different approaches to quantify the EOF in a nanopipette. First, we filled nanopipettes with PBS containing the zwitterionic and hence neutrally charged fluorescent dye rhodamine B^41^ and visualized the EOF (Figure 2a, top row) and, for comparison, the pressure-induced flow^20^ (Figure 2a, bottom row) by imaging the nanopipettes sideways with a fluorescent microscope. Without voltage or hydrostatic pressure applied, no fluid flowed out of the nanopipette (Figure 2a, left image). For an increasing applied voltage *V*_0_, the EOF progressively increased, visibly by more dye being released from the nanopipette (Figure 2a, top row), as expected from eq 2. Likewise, for a larger applied hydrostatic pressure *p*_0_, the pressure-induced flow progressively increased as expected (Figure 2a, bottom row). Visually comparing EOF and pressure-induced flow demonstrates that EOF and pressure-induced flow are comparable for a few volts of applied voltage and kilopascals of applied hydrostatic pressure, as already predicted in the previous section.

However, since imaging the nanopipette sideways is not practical in a SICM experiment, we used the streaming current to quantify the EO effect and specifically the EO mobility, as widely applied for capillaries and microchannels.^42^ The streaming current *I*_str_ in a conical nanopipette due to fluid flow induced by external pressure *p*_0_ can be described as^35^

$$I_{\mathrm{str}}=\mu_{\mathrm{eo}}\cdot \pi r_{i}\tan\alpha \cdot p_{0}$$ 5

As shown for nanopipettes of different sizes and treatments, the streaming current follows the expected linear dependency on the applied pressure (Figure 2b). The slope, *I*_str_/*p*_0_, sometimes denoted as streaming conductance, is positive, as expected, for the negatively charged bare glass with *μ*_eo_ > 0 and is larger for the large nanopipette compared to the small nanopipette. The BSA-treated nanopipette shows an even smaller but still positive streaming conductance, as BSA partly passivates the negative surface charge of the glass. In contrast, the PLL-treated nanopipette shows a negative streaming conductance, as PLL makes the nanopipette surface positively charged and hence *μ*_eo_ < 0.

To calculate the EO mobility independent of nanopipette geometry, we separately measured the ion current *I*_0_ though the nanopipette induced by an externally applied voltage *V*_0_ in the absence of external pressure, described as *I*_0_ = σ·π*r*_*i*_ tan α·*V*_0_.^37^ Combined with eq 5 this gives the EO mobility as

6 $$\mu_{\mathrm{eo}}=\frac{I_{\mathrm{str}}}{p_{0}}\cdot \frac{\sigma V_{0}}{I_{0}}$$

We measured the streaming current and the ion current of several nanopipettes of very different sizes with opening radii between about 50 and 500 nm. Although the streaming conductance and ion current strongly varied depending on the nanopipette size (not shown), the EO mobilities calculated from eq 6 were in a narrow range (Figure 2c, left axis). For bare glass, *μ*_eo_ was measured as about 20·10^–9^ m^2^/(V s), in accordance with the literature.^31^ The EO mobility for BSA-treated nanopipettes was lower (about 10·10^–9^ m^2^/(V s)), but still positive, also in accordance with the literature,^43^ and that for PLL-treated nanopipettes was negative (Figure 2c, bars). Repeating the measurements with 5 to 14 individual nanopipettes per condition showed that *μ*_eo_ was measured reproducibly, typically within a variation of about 10%, for bare and BSA-treated glass (Figure 2c, markers). For PLL-treated nanopipettes, the variation was larger (within about 50%), probably due to variations in coating efficiency, as the electrokinetic properties strongly depend on the PLL coverage.^44^ As *μ*_eo_ is directly related to the (negative) ζ potential, rearranging eq 1 allows to also quantify the ζ potential of the glass surfaces (Figure 2c, right axis); the obtained values were consequently also in accordance with the literature.^31,43,44^ In conclusion, the streaming current was used to reproducibly and accurately measure *μ*_eo_ and was therefore used in the following to “calibrate” the EO effect for each nanopipette in the experiments before approaching the nanopipette to the sample.

### Quantifying the EOF on a Decane Microdroplet

To investigate the effect of EOF on sample deformation, a decane microdroplet was imaged with the SICM (Figure 3a). The absolute ion current increased linearly with the applied voltage, as expected (Figure S1a,b), and the ion current measurement was generally stable with a current noise of 1–2 pA, weakly increasing with the applied voltage (Figure S1c). Consequently, SICM imaging was stable for applied voltages of up to at least 2500 mV (Figure S1d,e).

**Figure 3:**
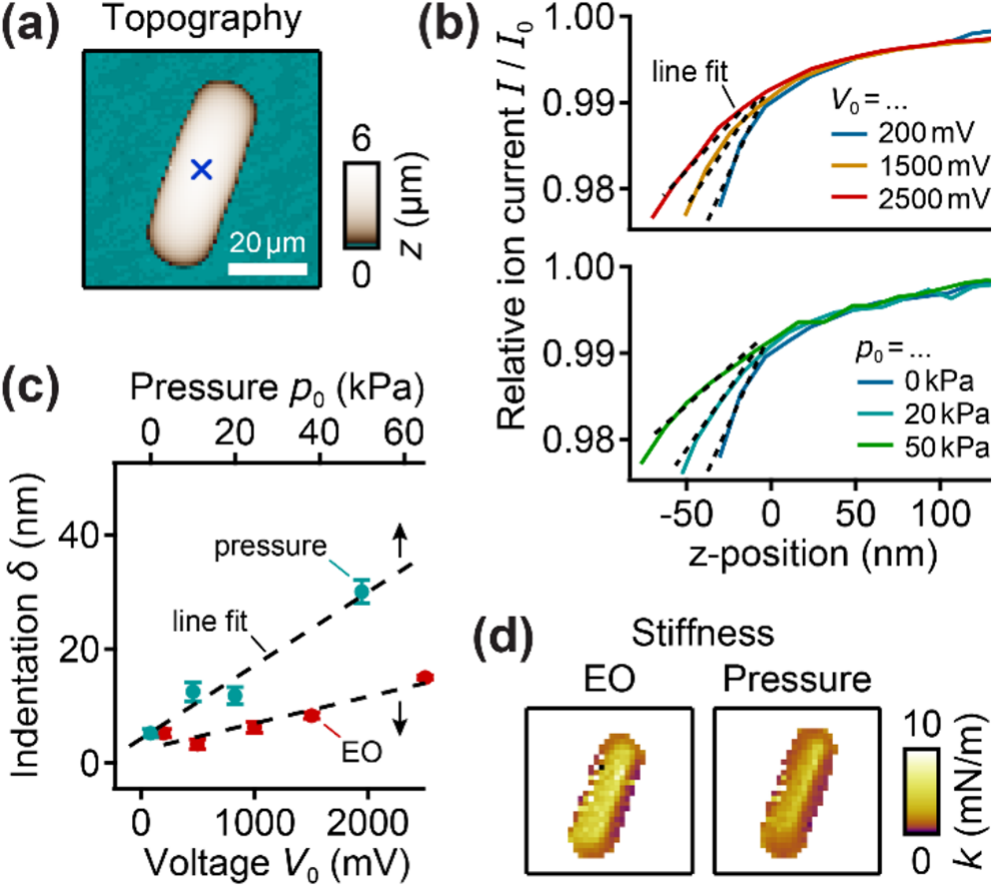
Quantifying EOF on a decane droplet. (a) Representative topography image of the decane droplet. (b) *IZ*-curves recorded using a small nanopipette (*r*_i_ = 50 nm) with different applied voltages *V*_0_ (top) or pressures *p*_0_ (bottom) with line fits. Location marked in (a) with blue cross. (c) Indentation δ as a function of applied pressure *p*_0_ and voltage *V*_0_ with line fits. Markers show median and error bars indicate median absolute deviation (MAD). (d) Stiffness maps of the droplet recorded with EOF using *V*_0_ = 2500 mV (left) and with pressure using *p*_0_ = 50 kPa (right).

As shown by exemplary *IZ*-curves recorded with a small nanopipette (*r*_i_ = 50 nm) on the top of the droplet (Figure 3b), the droplet was increasingly indented for a larger applied voltage *V*_0_ (Figure 3b, top). Likewise, when applying hydrostatic pressure (and only a small voltage *V*_0_ = 200 mV), the droplet was also increasingly indented for a larger applied pressure *p*_0_ (Figure 3b, bottom).

We quantified the indentation using line fits to the *IZ*-curves (Figure 3b, dashed lines) as δ = 0.01­(*s* ^–1^ – *s*_∞_^–1^), where *s* is the slope of the *IZ*-curve on the droplet between 98 and 99% ion current and *s*_∞_ is the slope of the *IZ*-curve on an infinitely stiff sample.^7,22^ The indentation increased linearly with increasing applied voltage *V*_0_ (Figure 3c, red markers), since the effective EO pressure *p*_eo_ is proportional to *V*_0_ as predicted from eq 4. Furthermore, the larger the nanopipette, the smaller *p*_eo_ should be. We hence repeated the measurements with a medium (Figure S2a) and a large (Figure S2b) nanopipette, where the indentation by the EOF was smaller (Figure S2a, red markers) and negligible (Figure S2b, red markers), respectively.

When applying hydrostatic pressure, the indentation also increased linearly with increasing pressure (Figure 3c, blue markers), as shown earlier.^7^ By comparing applied pressure and voltage for a given indentation, we estimated the effective EO pressure (Figure S2c), which well follows the expected dependency with *r*_i_^–2^ (Figure S2c, inset). Assuming that the effective EO pressure is equivalent to *p*_eo_ and fitting eq 4 (Figure S2c, dashed curve) yields *μ*_eo_ *η* ≈ 1.4·10^–11^ m^2^Pa/V, slightly lower than the theoretical value for glass and water (≈ 2·10^–11^ m^2^Pa/V, see above). Note that a lower value is expected since the assumptions used to derive eq 4 are not fully fulfilled in the experiment, as explained in the following.

Next, we aimed to use the EOF to quantify the mechanical stiffness of the decane microdroplet (Figure 3d) by developing a theoretical model, as described in the following.

### Numerical Model for EOF in a Nanopipette

The situation in the SICM experiment is more complex than in the theoretical considerations above (see Results and Discussion, first section), because for typical pipet-sample distances the EOF is usually not completely blocked by the sample and the applied voltage *V*_0_ only partially drops along the nanopipette. To account for these aspects, we first developed a numerical FEM model to calculate the EOF in a SICM nanopipette and the resulting deformation of an elastic sample with Young’s modulus *E* (Figure 4, see Methods and Figure S3a for details). For a large pipet-sample distance, the EOF almost freely exits the nanopipette and hardly deforms the elastic sample (Figure 4a, top). For a small pipet-sample distance, the EOF is partly blocked by the sample and the pressure below the nanopipette opening increases (Figure 4a, bottom) as predicted above (Figure 1c). As expected, the effective pressure on the sample surface generated by EO is slightly smaller than the theoretical maximum pressure *p*_eo_ given by eq 4, because the EOF is not fully blocked by the sample.

**Figure 4:**
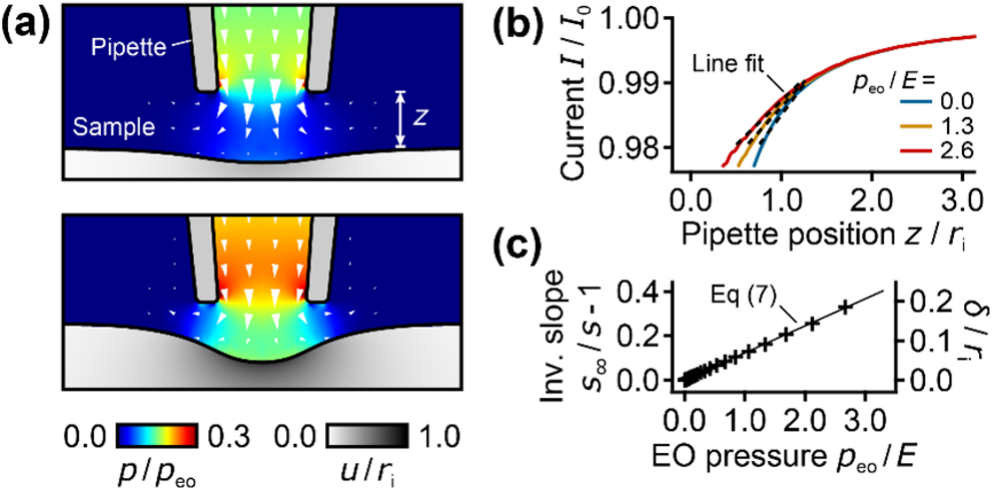
FEM model for EOF deforming an elastic sample. (a) FEM results for fluid pressure (color), deformation (greyscale), and flow velocity (arrows) for a large (top) and a small (bottom) pipette-surface distance *z* (in units of *r*_i_). (b) Simulated *IZ*-curves for different ratios of EO pressure *p*_eo_ to sample Young’s modulus *E* with line fits. (b) Inverse slope, *s*_∞_/*s* – 1, or sample indentation δ as a function of the ratio *p*_eo_/*E* with fit of eq 7. Parameters for simulations shown here: α = 4°, *p*_eo_/*E* = 2.6, *z* = 1.3*r*_i_ (top, corresponds to *I*/*I*_0_ = 99% relative ion current) and *z* = 0.8*r*_i_ (bottom, 98%).

Next, we simulated *IZ*-curves for different ratios of EO pressure *p*_eo_ to sample Young’s modulus *E* (Figure 4b). Quantifying the sample indentation from the slope *s* of the *IZ*-curve between 98 and 99% ion current (Figure 4b, line fits) shows that the inverse slope, *s*_∞_/*s* – 1, linearly increases with the ratio *p*_eo_/*E* (Figure 4c, black markers). Hence, similar to our previous model,^22^ the Young’s modulus *E* of the sample follows from the slope *s* of the *IZ*-curve with EOF as

7 $$E\left(s\right)=A_{\mathrm{eo}}\cdot p_{\mathrm{eo}}{\left(\frac{s_{\infty }}{s}-1\right)}^{-1}$$

where *A*_eo_ is a geometrical parameter, *s*_∞_ the slope of the *IZ*-curve on an infinitely stiff sample, and *p*_eo_ the EO pressure given by eq 4. *A*_eo_ is a unitless parameter weakly depending on the nanopipette geometry, mainly the inner half cone angle. For a given nanopipette geometry, we determined *A*_eo_ by fitting eq 7 to the inverse slope of the *IZ*-curve vs the ratio *p*_eo_/*E* (Figure 4c, black curve), as tabulated in Table S1.

Additionally, since samples such as the decane microdroplet behave like Hookean springs,^7^ we also developed a model for a flat sample with a stiffness in terms of a spring constant *k* (see Figure S3a–c for details). Again, the EOF is increasingly blocked and the pressure on the sample increases for a decreasing pipette-sample distance (Figure S3b), approaching *p*_eo_ as estimated by eq 4. We generated *IZ*-curves for different ratios of EO pressure to sample stiffness (Figure S3c). Analog to eq 7 and based on our other previous model,^7^ we relate the spring constant *k* to the slope *s* of the *IZ*-curve between 98 and 99% ion current (Figure S3d) as

8 $$k\left(s\right)=B_{\mathrm{eo}}\cdot p_{\mathrm{eo}}r_{i}{\left(\frac{s_{\infty }}{s}-1\right)}^{-1}$$

where *B*_eo_ is a geometrical parameter weakly depending on the pipette shape, mainly the inner half cone angle α (Table S1). As in our previous model,^7^ due to the same functional form of eqs 7 and 8, (effective) stiffness and (apparent) Young’s modulus are proportional to each other with *k*/*E* = *r*_i_ *B*_eo_/*A*_eo_ ≈ 10*r*_i_.

### Measuring the Stiffness of a Decane Microdroplet with EOF

As a first application, we mapped the mechanical stiffness of the decane microdroplet investigated above (Figure 3a). The stiffness of the droplet measured with EOF using *V*_0_ = 2500 mV (corresponds to *p*_eo_ ≅ 64 kPa) is relatively homogeneous with about 5 mN/m on the top of the droplet and slightly lower on the sides due to the slope of the droplet surface (Figure 3d, left). In comparison, the stiffness map measured with a hydrostatic pressure of *p*_0_ = 50 kPa (and only a small voltage of *V*_0_ = 200 mV) is qualitatively and quantitatively very similar (Figure 3d, right). This shows that both EOF and hydrostatic pressure can be used to accurately measure the stiffness of elastic samples.

### Mapping the Young’s Modulus of Living Cells with EOF

As a second application, we mapped the Young’s modulus of living cells with the SICM using EOF. First, we investigated live U2OS osteosarcoma cells stably transfected with RFP-tagged actin showing a well-defined actin cytoskeleton (Figure 5a) with pronounced stress fibers in the lamellipodium (Figure 5a, zoom-in). When imaging the same region with the SICM and mapping the Young’s modulus with EOF using a voltage of *V*_0_ = 900 mV (corresponds to *p*_eo_ ≅ 5.3 kPa), the Young’s modulus map shows the same stress fibers with *E* ≈ 1 kPa and the soft cytosol with *E* ≈ 0.2 kPa (Figure 5b).

**Figure 5:**
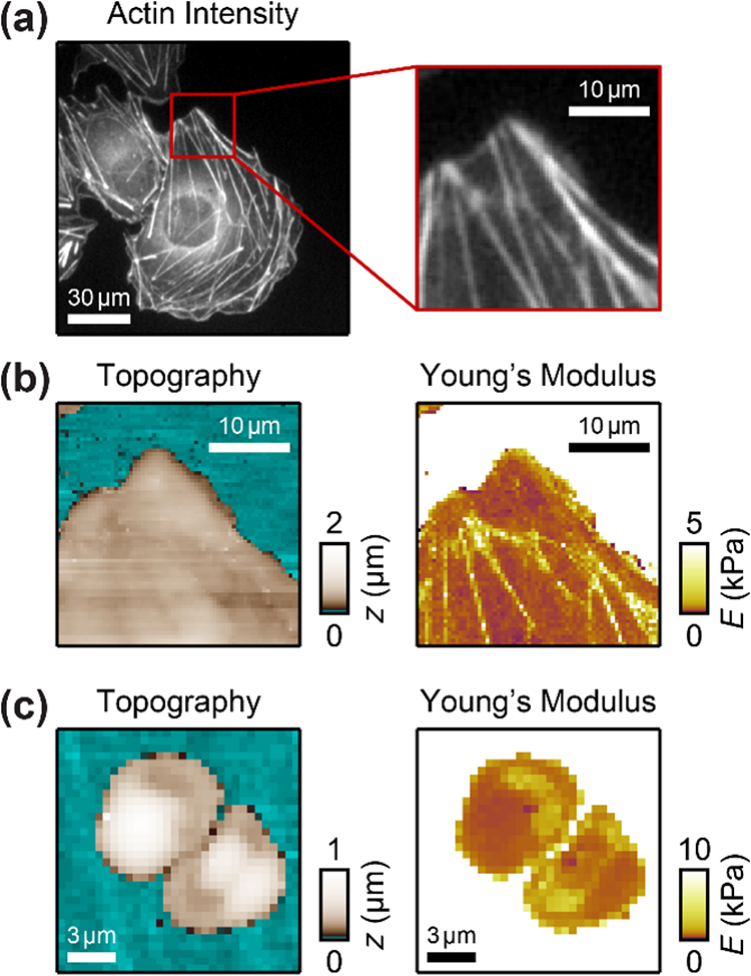
Application of EOF to map the Young’s modulus of living cells. (a) Actin fluorescence image with zoom-in of the lamellipodium of a living U2OS and (b) SICM topography image and Young’s modulus map of the same region measured with EOF using a voltage of *V*_0_ = 900 mV. (c) SICM topography image and Young’s modulus map of two living human platelets measured with EO using a voltage of *V*_0_ = 700 mV.

We also imaged living human thrombocytes (platelets) with the SICM and measured their Young’s modulus with EOF using a voltage of *V*_0_ = 700 mV (corresponds to *p*_eo_ ≅ 11.2 kPa) (Figure 5c). The stiffer regions had *E* ≈ 5 kPa and the softer regions *E* ≈ 1 kPa.

For comparison, we imaged the U2OS cell and platelets also with hydrostatic pressure (and only a small voltage *V*_0_) (Figure S4). The Young’s modulus was qualitatively and quantitatively similar to that in Figure 5, showing that also on live cells both EOF and hydrostatic pressure can be used to accurately and consistently measure the local cellular Young’s modulus with submicrometer resolution.

## Summary and Conclusion

In this manuscript, we investigated the mechanical effects of EOF in SICM experiments (Figure 1). By analytical considerations and experimentally we showed that the fluid flow by EOF is comparable with that of the classically applied hydrostatic pressure of a few kilopascals for glass nanopipettes with typical opening radii below 100 nm and/or applied voltages above 1 V (see eq 4 and Figure 2a). Since the EO mobility depends on various experimental conditions such as electrolyte composition, pH, or temperature,^34^ we also introduced the streaming current (Figure 2b) to reproducibly quantify the EO mobility and ζ potential of nanopipettes in a running SICM experiment (Figure 2c). We then demonstrated that EOF can be used to deform elastic samples such as decane microdroplets (Figure 3), where we found the expected linear dependency of the EO effect on the applied voltage as well as the expected inverse quadratic dependency with the nanopipette opening radius as 1/*r*_i_^2^. Even though the applied voltages partly exceed the electrolysis limit of typically ± 1 V for Ag/AgCl electrodes in aqueous solutions,^45^ we did not observe any gas bubble formation, and the ion current measurement and SICM imaging were stable up to voltages of at least 2500 mV (Figure S1). The use of, for example, salt bridges could further increase the ion current stability.^46^ As the geometry of the nanopipette-sample system is rather complex, we developed a numerical model to quantify the stiffness of an elastic sample from the slope of an *IZ*-curve with EOF (Figures 4 and S3) and use it to quantitatively measure the stiffness of a decane microdroplet. As further applications, we mapped the Young’s modulus of living human U2OS osteosarcoma cells and thrombocytes with the SICM using EOF (Figure 5), giving results qualitatively and quantitatively similar to the established hydrostatic pressure method (Figure S4). The selection of the applied voltage is, however, not trivial, as the EOF depends on various further experimental conditions, specifically electroosmotic mobility, electrolyte viscosity, and nanopipette opening radius. One strategy could be, possibly after quantifying the electroosmotic mobility using the streaming current, to choose the applied voltage so that *p*_eo_ according to eq 4 is comparable to the expected (apparent) Young’s modulus of the sample, similar as for the selection of *p*_0_ in the hydrostatic pressure method.^22^

In SICM experiments, the applied voltage is usually relatively small, typically a few hundred millivolts up to 1 V, depending on the required signal-to-noise ratio,^23^ but the EO effect can become large for small nanopipettes due to the quadratic dependency on the nanopipette opening radius as *p*_eo_ ∝ 1/*r*_i_^2^. Such a situation might occur in SICM experiments employing the “intrinsic colloidal pressure” method, where nanopipettes with opening diameters less than 100 nm are used to exert small forces on the sample to quantify the sample elastic modulus;^47,48^ and the pressure caused by the EOF then adds to the other interaction forces acting on the sample. Additional pressure effects can arise from the weight^49^ and/or the capillary action^32^ of the water column in the nanopipette capillary, but these effects are comparably small (on the order of a few hundred Pascals). In general, if hydrostatic pressure is additionally applied to the nanopipette, the effects of EOF and pressure-induced flow simply add due to the linearity of the underlying equations as confirmed by FEM (not shown).

EOF in the nanopipette might also be an explanation for the fact that in practice in the SICM usually positive voltages are applied to the nanopipette electrode relative to the bath electrode, as the then outflowing EOF might reduce the risk of contaminations from the sample entering the nanopipette.^50^ For simplicity, we have not included a possible surface charge of the sample in our model, which can affect the EOF for small tip–sample distances^51^ or in an asymmetric-conductivity configuration.^52^

In summary, EOF in the nanopipette can be used for mechanical SICM measurement and might be a relevant aspect in SICM experiments in general, especially when using high voltages and/or small nanopipettes with opening radii on the order of 100 nm or below. The effect of EOF can be estimated by the resulting EO pressure and can be used to mechanically probe soft samples. Hence, EOF might be a valuable extension to the SICM methodology.

## Supplementary Material



## Data Availability

The data generated and analyzed in the present study are available from the corresponding author upon request.

## Notes

Stability of the current measurement and of SICM imaging at high voltages (Figure S1); dependency of the EO pressure on the nanopipette size (Figure S2); parameters for eqs 7 and 8 for different values of the nanopipette inner half cone angle α calculated by FEM (Table S1); schematic of FEM model and model for a flat sample (Figure S3); comparison with Young’s modulus from hydrostatic pressure (Figure S4) (PDF)
