# Supplementary material for: Quantifying and Utilizing Electroosmotic Flow for Mechanical Measurements with the Scanning Ion Conductance Microscope
Source: Anal Chem. 2025 Oct 3;97(41):22541–7. doi: 10.1021/acs.analchem.5c03186 (PMC12547853; doi:10.1021/acs.analchem.5c03186)
Supplement: Supplementary file 1 [file ac5c03186_si_001.pdf]

Supporting Information

Quantifying and Utilizing Electroosmotic Flow for Mechanical Measurements with the Scanning Ion Conductance Microscope

Johannes Rheinlaender* and Tilman E. Schäffer

Institute of Applied Physics, University of Tübingen

Auf der Morgenstelle 10, 72076 Tübingen, Germany

*E-Mail:johannes.rheinlaender@uni-tuebingen.de

Table of Contents

Figure S1 2

Figure S2 2

Table S1 2

Figure S3 3

Figure S4 3

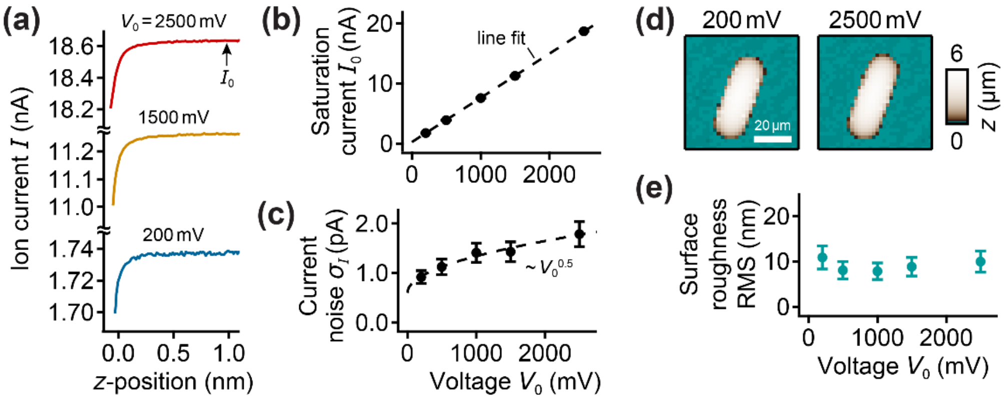

Figure S1. Stability of the current measurement and of SICM imaging at high voltages. (a) *IZ*-curves for different applied voltages from Figure 3b (upper panel) shown as absolute current. **(b)** Saturation current I_0 and **(c)** current noise σ_I vs. applied voltage V_0, showing the expected linear and square root dependencies, respectively. **(d)** Representative topography images of the decane droplet from Figure 3 and **(e)** apparent substrate roughness showing that the SICM imaging quality did not depend notably on the applied voltage. Markers show average (b), standard deviation (c), or RMS values (e) and error bars indicate standard errors.

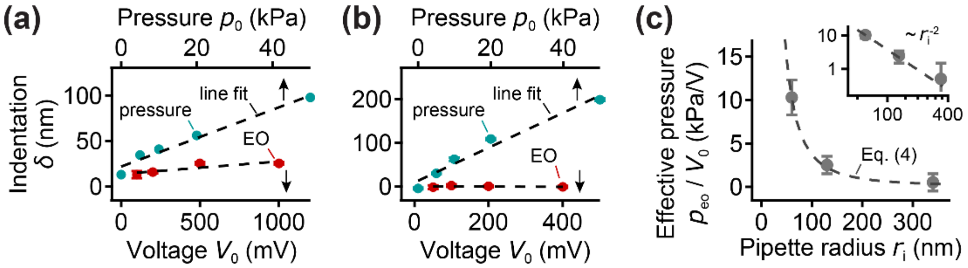

Figure S2. Dependency of the EO pressure on the nanopipette size. (a) Indentation δ of the decane droplet shown in Figure 3 as a function of applied pressure p_0 and voltage V_0 for a medium nanopipette (r_i = 130 nm) and **(b)** a large nanopipette (r_i = 340 nm) and with line fits. Markers show median and error bars indicate MAD. **(c)** Effective pressure p_{eo}/V_0 for the three different nanopipettes as a function of their pipette opening radius r_i with fit of Equation (4). Inset shows data as log-log plot illustrating dependency with r_i^{-2}.

Table S1. Parameters for Equations (7) and (8) for different values of the nanopipette inner half cone angle α calculated by FEM (see Figure 4 and Figure S2 for details).

Inner half cone angle α	2°	3°	4°	5°	6°
Infinite stiffness slope s_∞ (I_0/r_i)	0.027	0.021	0.018	0.016	0.014
Geometrical parameter A_{eo}	0.33	0.24	0.12	0.090	0.071
Geometrical parameter B_{eo}	1.4	0.90	0.71	0.56	0.45

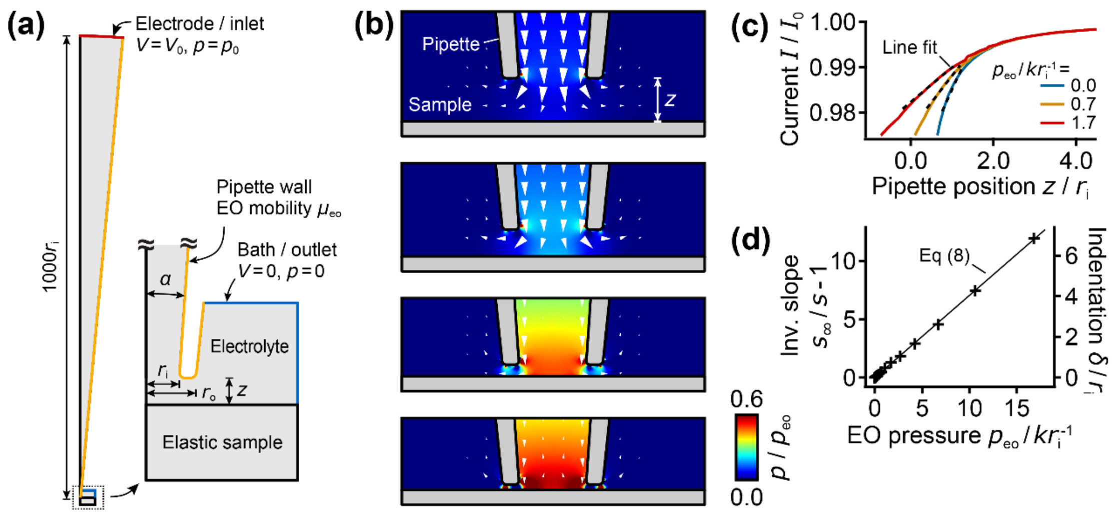

Figure S3. Schematic of FEM model and model for a flat sample. (a) Schematic of the FEM model for simulating EOF (and optionally pressure-induced flow) through a SICM nanopipette. **(b)** FEM model for EOF through a pipette close to a flat, stiff sample for different pipette-sample distances z ($z = 1.3r_\mathrm{i}$, corresponding to $I/I_0 = 99\%$ relative ion current; $z = 0.8r_\mathrm{i}$, 98%; $z = 0.4r_\mathrm{i}$, 95%; $z = 0.2r_\mathrm{i}$, 90%). The arrows indicate fluid flow velocity, and the color plots indicate fluid pressure in units of the EO pressure p_eo. **(c)** IZ-curves simulated for different ratios of EO pressure to sample stiffness (ratio p_eo to k/r_i). **(d)** Inverse slope of simulated IZ-curves, $s_\infty/s - 1$, or indentation δ as a function of the ratio of EO pressure p_eo to sample stiffness in units of k/r_i with fit of Equation (8).

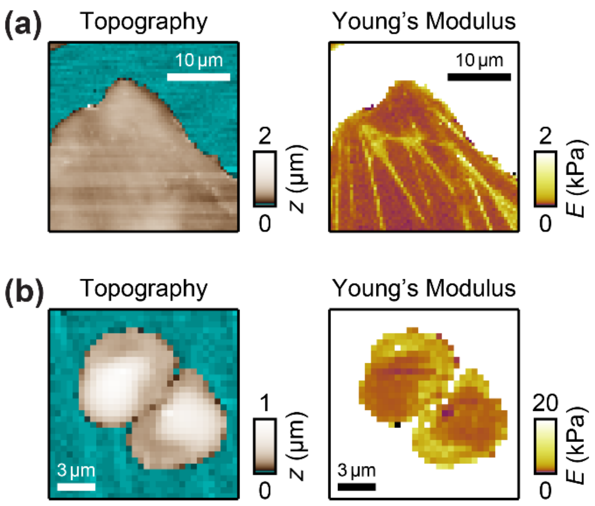

Figure S4. Comparison with Young's modulus from hydrostatic pressure application. (a) SICM topography images and Young's modulus maps of the lamellipodium of the living U2OS cell shown in Figure 5b and **(b)** of the two living human platelets shown in Figure 5c as measured with hydrostatic pressures of $p_0 = 5$ kPa and 10 kPa, respectively.
